# Herbal Medicine: High-Test Mothers’ Milk

**DOI:** 10.1289/ehp.114-a344

**Published:** 2006-06

**Authors:** Cynthia Washam

A lack of regulation means that herbal remedies can be ineffective or, conversely, far more potent than users may suspect. Researchers in Taiwan have found a new reason for consumers to be wary of certain herbal remedies: some herbs used in traditional Chinese medicine contain lead, which mothers can pass to their infants through breast milk. The study, published in the 1 February 2006 issue of *Science of the Total Environment*, adds to the growing evidence that infants can be exposed to potentially dangerous lead levels *in utero* and through breast milk.

Bans on leaded paint and gasoline have caused lead exposure during childhood to plummet. Now scientists are focusing more attention on perinatal exposure to lead via maternal circulating blood levels and breast milk. The neurotoxicant is associated with behavioral problems and diminished intelligence. Although lead has declined in the environment in a number of countries, it may still persist in soil, dust, and water in many parts of the world. Maternal bone lead accumulated during earlier exposure is released during pregnancy and lactation as the body redistributes its calcium stores.

Principal investigator Bor-Cheng Han and colleagues originally recruited 72 pregnant women, but only 16 completed the study. The women were interviewed during pregnancy and lactation to collect information on residential and occupational lead exposures, socio-demographic characteristics, and consumption of nutritional supplements, traditional Chinese herbs, alcohol, and tobacco. The women provided breast milk samples weekly from 1 to 60 days postpartum. Nine of the women took traditional herbs, while seven did not.

The researchers purchased samples of herbs that the mothers reported taking regularly—*Angelicae sinensis radix*, *Lycii fructus*, *Zizyphi fructus*, and a preparation known as Shy-Wuh-Tang. Then they tested the samples for lead content. All the samples contained lead; Shy-Wuh-Tang, used to treat menstrual and circulatory problems, had the highest levels, at 322.31 micrograms per kilogram (μg/kg).

The nine herb users had a mean lead concentration of 9.94 μg per liter (L) in colostrum, the form of milk produced just after delivery. Lead levels in their breast milk dropped with most weekly samplings, to a mean concentration at the final sampling of 2.34 μg/L. Lead levels also declined in the seven mothers not using herbs, from 8.11 μg/L in colostrum (likely reflecting occupational or pollution exposures) to 2.36 μg/L in mature milk.

The finding that Chinese herbs were contaminated with lead comes as no surprise to research scientist Richard Ko of the California Department of Health Services. In the 17 September 1998 issue of the *New England Journal of Medicine*, Ko reported high lead levels in 24 of 260 Chinese patent medicines sold in California. Tainted products slip easily into the hands of consumers because the FDA does not have enough resources to inspect all imported herbs, nor does it regulate herbs, which it considers dietary supplements rather than drugs. The Taiwanese researchers speculate that the herbs their subjects took were grown in contaminated soil.

In spite of the risks, the worldwide market for herbal treatments is estimated to be more than $60 billion and growing fast, according to the UN Conference on Trade and Development. Some 30–50% of all medicines consumed in China are traditional herbs.

It is unclear how much risk the lead-contaminated herbs posed to the babies in the Taiwan study. Jenny Pronczuk de Garbino, a physician with the WHO Department of Public Health and the Environment, says that “only if the doses were extremely high would they outweigh the benefits of breastfeeding,” but that “prevention of exposure is paramount.” The FAO/WHO Joint Expert Committee on Food Additives and Contaminants, an independent scientific expert body regularly convened by the FAO and the WHO, last assessed the risk of lead exposure to human health in 1999, and established a provisional tolerable weekly intake of 25 μg/kg body weight as a value that would not lead to any appreciable health risk. The WHO maintains in its 2003 document *Global Strategy for Infant and Young Child Feeding* that “[b]reastfeeding is an unequalled way of providing ideal food for the healthy growth and development of infants.”

Han admits his study of just 16 mothers is too small to draw conclusions from. But it adds to the growing information about how mothers’ exposure may influence their infants’ lead levels. The Lead and Pregnancy Work Group organized by the CDC is reviewing such studies in an effort to develop national guidelines on assessing and managing risk of lead exposure during pregnancy and lactation.

“We need a better understanding of neonatal exposure from breastfeeding,” says Adrienne Ettinger, a Harvard School of Public Health researcher and member of the CDC work group. “We don’t have all the scientific data yet.”

## Figures and Tables

**Figure:**
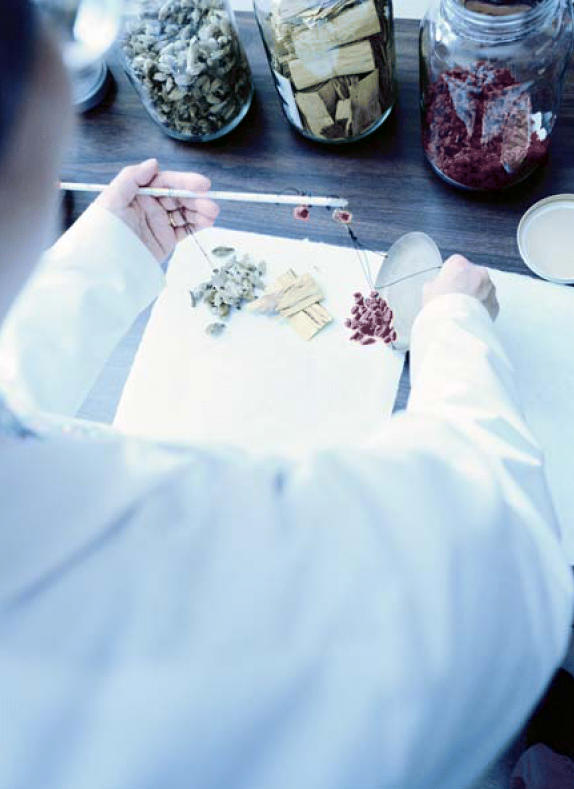
Bad medicine? Testing revealed elevated lead in the breast milk of mothers who took four traditional Chinese herbal remedies.

